# Supplementary material for: Evolutionary dynamics of the arthropod molting machinery
Source: Mol Biol Evol. 2026 Jul 27;43(8):msag181. doi: 10.1093/molbev/msag181 (PMC13449613; doi:10.1093/molbev/msag181)
Supplement: Supplementary file 1 [file msag181_supplementary_data.zip › Campli-Moulting-Genes-SUPPLEMENTARY-TABLES_v2.pdf]

Supplementary Tables

Evolutionary dynamics of the arthropod moulting machinery

Giulia Campli[1,2], Sagane Joye[1,2], Olga Volovych[3], Asya V. Novikova[3], Ariel D. Chipman[3], Marc Robinson-Rechavi[1,2], Robert M. Waterhouse[2]

[1] Department of Ecology and Evolution, University of Lausanne, 1015, Lausanne, Switzerland.

[2] Swiss Institute of Bioinformatics, 1015, Lausanne, Switzerland.

[3] The Department of Ecology, Evolution and Behaviour, The Hebrew University of Jerusalem, Edmond J. Safra Campus, Givat Ram, Jerusalem, 9190401, Israel.

CONTENTS

Supplementary Table 1: Genomic resources... 3
Supplementary Table 2: Metrics of the concatenated super-alignment used for species phylogeny reconstruction..4
Supplementary Table 3: Time estimates for species phylogeny tree calibration...............................5
Supplementary Table 4: Curated list of genes involved in the moulting process.............................. 8
Supplementary Table 5: Summary of deep time events from selected gene families.......................9
Supplementary Table 6: Query genes for gene prediction in unannotated assemblies.................. 10

Assembly Accession	TaxId	Species	Arthropoda Complete BUSCO	Arthropoda Single BUSCO	Arthropoda Duplicated BUSCO	Number of protein coding genes	Arthropoda Complete BUSCO Proteomes	Arthropoda Single BUSCO Proteomes	Arthropoda Duplicated BUSCO Proteomes
GCA_000376725.2	123851	Ladona fulva	93.7	92.3	1.4	18816	78.2	76.6	1.6
GCA_000507165.2	1049336	Ephemera danica	94.5	91.6	2.9	18562	85.8	84.3	1.5
GCA_000611955.2	407821	Stegodyphus mimosarum	93.7	90.2	3.5	27135	80	78.1	1.9
GCA_000767015.2	6954	Dermatophagoides farinae	84.8	83.6	1.2	15208	90	88.4	1.6
GCA_000828355.1	52283	Sarcoptes scabiei	84.9	84.7	0.2	10473	80.9	80.5	0.4
GCA_001187945.1	7375	Lucilia cuprina	98.6	97.6	1	14452	91	90	1
GCA_001718145.1	48709	Orchesella cincta	93	88.4	4.6	20247	90	85.2	4.8
GCA_002081605.1	418985	Tropilaelaps mercedesae	89.6	87.9	1.7	14303	85.9	84.1	1.8
GCA_003018175.1	6973	Blattella germanica	96.5	94	2.5	28676	84.9	82.9	2
GCA_003335185.2	195883	Laodelphax striatellus	97	93.4	3.6	17512	95	90.9	4.1
GCA_003675905.2	299467	Leptotrombidium deliense	86	84.9	1.1	14667	83.2	82.2	1
GCA_003675995.1	1965070	Dinothrombium tinctorium	93.6	54.3	39.3	19024	88.5	54.4	34.1
GCA_005959815.1	7385	Sarcophaga bullata	96.8	94.9	1.9	15763	87.8	86.2	1.6
GCA_006783055.1	1923959	Trinorchestia longiramus	86.4	83.1	3.3	26080	86.5	83	3.5
GCA_007210705.1	6832	Tigriopus californicus	93.5	92.8	0.7	15577	91.9	90.6	1.3
GCA_009176605.1	96803	Armadillidium nasatum	89.3	83.1	6.2	14636	75.3	70.5	4.8
GCA_009739505.2	248454	Apolygus lucorum	96.5	91.9	4.6	20111	94.3	89.8	4.5
GCA_009805615.1	1232801	Amphibalanus amphitrite	92.5	64	28.5	25581	85.2	61.3	23.9
GCA_011009095.1	2038154	Ignelater luminosus	96.9	94.4	2.5	27558	94.5	92.1	2.4
GCA_013167095.2	1820382	Daphnia sinensis	98.5	96.3	2.2	16955	97.4	94.8	2.6
GCA_013340265.1	36987	Coptotermes formosanus	98.5	97.3	1.2	12983	90.1	88.7	1.4
GCA_013358835.2	34615	Ixodes persulcatus	88.2	84.1	4.1	28396	78.2	75.9	2.3
GCA_015342795.1	94029	Argiope bruennichi	92.7	89.4	3.3	23259	87.3	83.8	3.5
GCA_016920775.1	1564500	Bradysia odoriphaga	97.8	94.9	2.9	16206	95	92.2	2.8
GCA_018290095.1	319348	Polypedilum vanderplanki	98.7	96.9	1.8	17863	98.7	96.7	2
GCA_019049445.1	860918	Ampulex compressa	99.2	99.2	0	18896	97.3	97.1	0.2
GCA_019343175.1	931172	Oedothorax gibbosus	95.2	91.4	3.8	29717	95.7	91.6	4.1
GCA_019925095.2	765133	Dendrolimus kikuchii	97.6	96.2	1.4	15321	95.4	93.9	1.5
GCA_021650745.2	40697	Blomia tropicalis	90.3	88.9	1.4	16592	90.4	88.3	2.1
GCA_021730765.1	59818	Tyrophagus putrescentiae	82.3	78.5	3.8	23009	86.5	82.7	3.8
GCA_022750525.1	2874060	Halotydeus destructor	90.5	89	1.5	14583	89.5	88.1	1.4
GCA_024506315.2	121845	Diaphorina citri	92.1	89.2	2.9	26564	95.4	90.8	4.6
GCA_025370935.1	7141	Choristoneura fumiferana	97.4	91.2	6.2	16231	83.1	77.4	5.7
GCA_026979955.1	439358	Megalurothrips usitatus	97	96.2	0.8	11617	90.3	89.2	1.1
GCA_029955175.1	1586481	Exocentrus adspersus	99.5	99.2	0.3	16908	88.5	87.7	0.8
GCA_030143305.2	6943	Amblyomma americanum	85.5	70.9	14.6	34557	79.9	71	8.9
GCA_030220185.1	6984	Diploptera punctata	99.2	92.6	6.6	28414	86	81.5	4.5
GCA_032884065.1	6661	Artemia franciscana	89.4	81.5	7.9	20122	81.3	75.4	5.9
GCA_033782935.1	88211	Petrolisthes cinctipes	92	75.2	16.8	44511	94.4	77	17.4
GCA_034508575.1	1843537	Petrolisthes manimaculis	92.4	79.7	12.7	40296	95.1	82.1	13
GCA_037055365.1	468196	Polyplax serrata	98.7	98.7	0	14991	97.3	96.9	0.4
GCA_037179515.1	373956	Halocaridina rubra	88.5	86.2	2.3	25341	82.5	81.4	1.1
GCA_038050395.1	536013	Parthenolecanium corni	95	89.7	5.3	14951	86.4	82.4	4
GCA_038098605.1	2509291	Gryllus longicercus	99	90.3	8.7	14730	95.6	87.6	8
GCA_038387795.1	85552	Scylla paramamosain	95.1	93.5	1.6	20946	89.9	88.4	1.5
GCA_038502225.1	27406	Cherax quadricarinatus	88.8	86.9	1.9	17532	88.3	86.4	1.9
GCA_040020575.1	488301	Rhynocoris fuscipes	98.5	95.1	3.4	13362	86.6	82.9	3.7
GCA_040285465.1	328185	Menopon gallinae	98.8	98.2	0.6	10891	88.5	87.5	1
GCA_902825445.1	874455	Arctia plantaginis	98.8	97.9	0.9	18402	98.5	97.5	1
GCA_902829235.1	197152	Cloeon dipterum	97.8	95.4	2.4	16299	97	94.5	2.5
GCA_902850365.2	168631	Chilo suppressalis	98.9	98.2	0.7	11366	95.5	94.9	0.6
GCA_905338385.1	69355	Darwinula stevensoni	90.3	88.1	2.2	15334	84.5	82.5	2
GCA_905338405.1	399045	Notodromas monacha	86.2	81.1	5.1	13723	80.8	76.7	4.1
GCA_905368565.1	1979941	Medioppia subpectinata	85.4	75.8	9.6	23249	81.1	72.9	8.2
GCA_905397405.1	334625	Oppiella nova	85.1	75.4	9.7	23525	82	72.8	9.2
GCA_910591605.1	39272	Allacma fusca	91	89.2	1.8	47610	91.8	89.7	2.1
GCA_918026855.4	80249	Phaedon cochleariae	99.7	92.7	7	13141	96	89.7	6.3
GCA_918697745.1	27404	Daphnia galeata	95.2	94.2	1	15649	93.1	91.8	1.3
GCA_918797505.1	7038	Bemisia tabaci	98.5	96.1	2.4	14348	96.3	94.1	2.2
GCA_928085145.1	85310	Nezara viridula	98.4	95.8	2.6	15971	91.3	88.9	2.4
GCA_964023285.1	280406	Larinioides sclopetarius	98.5	93.8	4.7	22843	95.7	91	4.7
GCA_964058975.1	48144	Meganyctiphanes norvegica	75.7	71.7	4	42189	85.3	82	3.3
GCF_000001215.4	7227	Drosophila melanogaster	99.6	99.1	0.5	13986	99.9	99	0.9
GCF_000006295.1	121224	Pediculus humanus	97.1	96.9	0.2	10758	97	96.7	0.3
GCF_000239435.1	32264	Tetranychus urticae	90.1	84.2	5.9	11686	91.6	85.1	6.5
GCF_000255335.2	34638	Galendromus occidentalis	93.9	90.8	3.1	11566	96.1	92	4.1
GCF_000341935.2	211228	Cephus cinctus	99.2	98.9	0.3	11511	99.6	99.2	0.4
GCF_000365465.3	114398	Parasteatoda tepidariorum	97	93.2	3.8	19750	98.3	93.3	5
GCF_000517525.1	6850	Limulus polyphemus	92.3	78.9	13.4	22873	93.9	80.4	13.5
GCF_000648675.2	79782	Cimex lectularius	99.4	97.8	1.6	11949	99.5	98.2	1.3
GCF_000648695.1	166361	Onthophagus taurus	99.6	96.7	2.9	14537	99.8	96.2	3.6
GCF_000671375.1	218467	Centruroides sculpturatus	93.9	89.4	4.5	24591	93.7	88.4	5.3
GCF_000696155.1	136037	Zootermopsis nevadensis	98.9	98.5	0.4	12381	98.4	98.1	0.3
GCF_000697945.3	133901	Frankliniella occidentalis	98.3	97.3	1	16516	99.5	97.9	1.6
GCF_000699045.2	224129	Agrilus planipennis	97.7	94.1	3.6	13371	97.8	90.3	7.5
GCF_000764305.2	294128	Hyalella azteca	93.7	93	0.7	17163	95.6	94.6	1
GCF_001272555.1	178035	Dufourea novaeangliae	99.8	99.7	0.1	9844	99.7	99.5	0.2
GCF_001412225.1	110193	Nicrophorus vespilloides	99.5	98.3	1.2	12642	100	98.6	1.4

GCF_001412515.2	454923	Diachasma alloeum	99.5	99	0.5	12351	99.1	98.5	0.6
GCF_001594045.1	520822	Atta colombica	99.2	98.7	0.5	10316	99.3	98.7	0.6
GCF_001901225.1	6956	Dermatophagoides pteronyssinus	86	84.8	1.2	11184	92.1	90.7	1.4
GCF_002217175.1	158441	Folsomia candida	97.5	95.9	1.6	24221	97.9	96.1	1.8
GCF_002443255.1	109461	Varroa destructor	94.2	92.7	1.5	10260	95.2	93.4	1.8
GCF_002532875.1	62625	Varroa jacobsoni	94.2	92.8	1.4	10739	95.8	94.3	1.5
GCF_002706865.2	69820	Spodoptera litura	98.8	97.6	1.2	14745	99	97.6	1.4
GCF_002891405.2	105785	Cryptotermes secundus	98.2	96.4	1.8	13170	98.3	96.8	1.5
GCF_003254395.2	7460	Apis mellifera	99.8	99.8	0	9935	99.2	99	0.2
GCF_003426905.1	7515	Ctenocephalides felis	96	69.4	26.6	18878	95.8	58.8	37
GCF_003676215.2	43146	Rhopalosiphum maidis	98.6	93.8	4.8	12060	98.6	93	5.6
GCF_003789085.1	6689	Penaeus vannamei	85	78.1	6.9	24987	93.7	82.5	11.2
GCF_008802855.1	7054	Photinus pyralis	99.4	89.9	9.5	20647	98.8	85.7	13.1
GCF_009176525.2	265458	Contarinia nasturtii	97.6	96.2	1.4	14889	99.5	97.7	1.8
GCF_010614865.2	202533	Stegodyphus dumicola	84.1	80.9	3.2	20729	87.5	84.8	2.7
GCF_011947565.2	41117	Pollicipes pollicipes	90.9	69.1	21.8	20444	93.3	57.3	36
GCF_012274295.1	473952	Osmia lignaria	99.4	99.1	0.3	10556	99	98.6	0.4
GCF_012932325.1	161013	Thrips palmi	96.9	95.9	1	14332	97.5	96.4	1.1
GCF_013123115.1	935657	Colletes gigas	99.1	97.7	1.4	10168	99.5	98.1	1.4
GCF_013339695.2	34632	Rhipicephalus sanguineus	92.5	86.7	5.8	22510	97.2	89.9	7.3
GCF_013339725.1	6941	Rhipicephalus microplus	90.8	87	3.8	18598	95.1	90.6	4.5
GCF_014805625.1	7396	Glossina fuscipes	99.4	96.1	3.3	12387	99.7	96.5	3.2
GCF_014839805.1	7130	Manduca sexta	98.3	89.8	8.5	15967	99.3	89.1	10.2
GCF_015228065.1	6687	Penaeus monodon	83.7	81.6	2.1	24092	91.3	87.3	4
GCF_016920785.2	6945	Ixodes scapularis	90.5	86	4.5	26659	98.5	93.9	4.6
GCF_017591435.1	210409	Portunus trituberculatus	93.5	92.9	0.6	17292	97.2	96.3	0.9
GCF_018991925.1	6706	Homarus americanus	93.2	92.2	1	22368	97.6	96.3	1.3
GCF_019202785.1	139456	Penaeus chinensis	90.8	89.8	1	20076	95.8	94.5	1.3
GCF_019457755.1	32260	Venturia canescens	99.6	98.9	0.7	11831	99.5	98.2	1.3
GCF_020424385.1	6728	Procambarus clarkii	94.4	93.2	1.2	26417	98.3	96.5	1.8
GCF_020631705.1	35525	Daphnia magna	98.6	94.4	4.2	16891	97.8	91.7	6.1
GCF_021130785.1	197043	Homalodisca vitripennis	83.3	73.7	9.6	19904	91.9	80.1	11.8
GCF_021134715.1	6669	Daphnia pulex	98.1	97.9	0.2	15295	97.3	96.3	1
GCF_021155785.1	2872261	Neodiprion fabricii	99.4	99.1	0.3	11597	99.5	99.2	0.3
GCF_021234035.1	35523	Daphnia pulicaria	98.8	97.2	1.6	16865	97.8	95.2	2.6
GCF_021461385.2	274613	Schistocerca piceifrons	97.5	94.1	3.4	17490	98.9	95.7	3.2
GCF_021461395.2	7009	Schistocerca americana	98.2	96.2	2	17662	99.1	96.9	2.2
GCF_022539665.1	120202	Daphnia carinata	98.7	97.6	1.1	13404	97.9	96.2	1.7
GCF_022605725.1	2921223	Anthonomus grandis	99.8	97.7	2.1	13112	99.1	96.9	2.2
GCF_023375885.1	34620	Dermacentor andersoni	93.7	90.6	3.1	22938	98.6	95.8	2.8
GCF_023864275.1	274614	Schistocerca cancellata	98.1	95.4	2.7	16907	99.4	96.2	3.2
GCF_023864345.2	2023355	Schistocerca serialis	98	95.8	2.2	17237	99.1	97.2	1.9
GCF_023897955.1	7010	Schistocerca gregaria	98.3	94.1	4.2	19799	99.2	96	3.2
GCF_024362695.1	13191	Pectinophora gossypiella	98.8	97.2	1.6	14107	99.7	97.8	1.9
GCF_024364675.1	116153	Aethina tumida	99.8	98	1.8	13131	99.5	97.6	1.9
GCF_024763615.1	29031	Phlebotomus papatasi	95.6	94	1.6	11610	97.6	95.9	1.7
GCF_025091365.1	58002	Daktulosphaira vitifoliae	98.7	95.8	2.9	14650	98.5	95.1	3.4
GCF_028554725.1	28588	Zeugodacus cucurbitae	99.3	99	0.3	13888	99.4	98.7	0.7
GCF_029603195.1	2530218	Condylostylus longicornis	98.7	98	0.7	12227	98.9	97.4	1.5
GCF_030269925.1	7091	Bombyx mori	99.4	99	0.4	13459	99.2	98.5	0.7
GCF_030625045.1	688607	Achroia grisella	98.9	95.7	3.2	13873	99.9	96.5	3.4
GCF_031216515.1	2078957	Neocloeon triangulifer	97.8	96.9	0.9	12721	98.6	97	1.6
GCF_031307605.1	7070	Tribolium castaneum	99.8	99.4	0.4	12172	99.3	98.5	0.8
GCF_037043105.1	334116	Vanessa tameamea	98.9	98.4	0.5	11875	99.8	99.6	0.2
GCF_037126465.1	34597	Ornithodoros turicata	96.4	92.1	4.3	22416	98.3	93.1	5.2
GCF_905115235.1	343691	Hermetia illucens	98.8	98.4	0.4	14004	99.5	99	0.5
GCF_905147105.1	7116	Pieris brassicae	99.1	98.7	0.4	11885	99.6	99.3	0.3
GCF_905475395.1	189513	Chrysoperla carnea	98	97.4	0.6	12985	98	97	1
GCF_907165205.1	41139	Coccinella septempunctata	99.6	98.8	0.8	14769	99.7	98.3	1.4
GCF_910589235.1	7445	Vespa crabro	99.6	99.3	0.3	10204	99.5	98.9	0.6
GCF_912999745.1	76193	Papilio machaon	99	98.1	0.9	13379	99.8	98.8	1
GCF_932276165.1	51655	Plutella xylostella	99.3	98.8	0.5	13971	99.6	98.9	0.7
GCF_943734665.1	42839	Anopheles aquasalis	98.1	97.4	0.7	11511	99.6	98.4	1.2
GCF_963082655.1	35570	Stomoxys calcitrans	99.2	98.7	0.5	15178	99.5	99	0.5
PRJEB109773	41365	Scolopendra cingulata	98.3	96.2	2.1	17955	97.6	95.2	2.4
PRJEB109799	29022	Scutigera coleoptrata	97.1	96.1	1	18157	98.1	97	1.1
PRJEB109837	2973175	Archispirostreptus syriacus	96.5	94.2	2.3	11437	95.1	92.6	2.5

Supplementary Table 1: Genomic resources.

Assembly accessions, protein-coding gene counts, and BUSCO assessments from *de novo* sequenced myriapod species and the publicly available annotated proteomes from NCBI.

Metric	Untrimmed	strictplus	automated1
Number of sequences in the alignment	145	145	145
Number of sites in the alignment	275927	76471	87877
Pairs of sequences	10440	10440	10440
Completeness (C) score for the alignment (Ca)	0.334825	0.772771	0.78738
Maximum C-score for individual sequences (Cr_max)	0.426689	0.863412	0.879821
Minimum C-score for individual sequences (Cr_min)	0.121347	0.305554	0.312562
Maximum C-score for individual sites (Cc_max)	0.986207	0.986207	0.986207
Minimum C-score for individual sites (Cc_min)	0	0.00689655	0.00689655
Maximum C-score for pairs of sequences (Cij_max, i!=j)	0.379426	0.857488	0.873471
Minimum C-score for pairs of sequences (Cij_min, i!=j)	0.0665466	0.167057	0.172719

Supplementary Table 2: Metrics of the concatenated super-alignment used for species phylogeny reconstruction.

The species phylogeny was computed from single-copy orthologues found in at least 95% of the total set of 145 species, as detected by the BUSCO assessments performed for the A3Cat on the genome assemblies using the arthropoda_odb10 lineage dataset. A concatenated super-alignment was built from the 145 trimmed individual multiple protein sequence alignments. The table shows AliStat statistics for the untrimmed supermatrix, as well as the trimmed supermatrices using the strictplus and automated1 options with TrimAl. The strictplus supermatrix was selected for building the phylogeny (76’471 columns, 71’014 distinct patterns, 57’142 parsimony-informative, 9’412 singleton sites, 9’917 constant sites).

Last Common Ancestor	Min estimate	Max estimate
Chelicerata	511.8	632
Hexapoda	425.4	478.1
Insecta	370.9	451.7
PanCrustacea	501.8	580
Myriapoda	442	550.9
Chilopoda	411.7	442.7
Malacostraca	254.2	444
Branchiopoda	365.1	491.7
Hymenoptera,Lepidoptera	310.7	389.7
Root	536.6	600

Supplementary Table 3: Time estimates for species phylogeny tree calibration.

Divergence time estimates were sourced from the TimeTree v5 database (Kumar et al. 2022) to time-calibrate the molecular species tree.

TaxId	Gene name	Moulting pathway	NCBI prot ID
7227	Bftz-F1	Early_genes	NP_524143.2
7227	Blimp-1	Early_genes	NP_001261442.1
7227	crc	Early_genes	NP_524897.1
7227	E74	Early_genes	NP_730288.1
7227	E75	Early_genes	NP_730321.1
7227	E78	Early_genes	NP_524195.2
7227	Hnf4	Early_genes	NP_723413.1
7227	HR3	Early_genes	NP_001334718.1
7227	HR38	Early_genes	NP_477119.1
7227	HR39	Early_genes	NP_476932.1
7227	HR4	Early_genes	NP_001259161.1
7227	HR78	Early_genes	NP_730636.1
7227	HR96	Early_genes	NP_524493.1
7227	tll	Early_genes	NP_524596.1
7227	Atet	Ecdysteroid_pathway	NP_001097079.1
7227	cyp18a1	Ecdysteroid_pathway	NP_728191.1
7227	dib	Ecdysteroid_pathway	NP_524810.2
7227	ecd	Ecdysteroid_pathway	NP_647707.1
7227	EcI	Ecdysteroid_pathway	NP_648989.1
7227	EcR	Ecdysteroid_pathway	NP_724460.1
7227	gcn5	Ecdysteroid_pathway	NP_648586.2
7227	MSBP	Ecdysteroid_pathway	NP_573087.1
7227	nvd	Ecdysteroid_pathway	NP_001097670.1
7227	phm	Ecdysteroid_pathway	NP_573319.1
7227	sad	Ecdysteroid_pathway	NP_650123.1
7227	shd	Ecdysteroid_pathway	NP_001261843.1
7227	spo	Ecdysteroid_pathway	NP_647975.2
7227	spok	Ecdysteroid_pathway	NP_001104460.2
7227	SREBP	Ecdysteroid_pathway	NP_730449.1
7227	sro	Ecdysteroid_pathway	NP_651725.1
7227	Usp	Ecdysteroid_pathway	NP_476781.1
7227	Broad	Fate_genes	NP_001162638.2
7227	chinmo	Fate_genes	NP_001188680.1
7227	E93	Fate_genes	NP_001097865.1
7227	Kr-h1	Fate_genes	NP_477466.1
7227	Cda3	Late_genes	NP_609806.1
7227	Cda4	Late_genes	NP_728468.1
7227	Cda5	Late_genes	NP_722590.2
7227	Cda9	Late_genes	NP_611192.1
7227	Chs2	Late_genes	NP_524209.3
7227	Cht11	Late_genes	NP_572361.1
7227	Cht2	Late_genes	NP_477298.2
7227	Cht3	Late_genes	NP_001036422.1
7227	Cht4	Late_genes	NP_524962.2
7227	Cht5	Late_genes	NP_650314.1
7227	Cht6	Late_genes	NP_001245598.1
7227	Cht7	Late_genes	NP_647768.3
7227	Cht8	Late_genes	NP_611542.2
7227	Cht9	Late_genes	NP_611543.3

7227	FABP	Late_genes	NP_001027179.1
7227	kkv	Late_genes	NP_730928.2
7227	knk	Late_genes	NP_649981.1
7227	mco1	Late_genes	NP_609287.3
7227	PPO1	Late_genes	NP_476812.1
7227	PPO2	Late_genes	NP_610443.1
7227	PPO3	Late_genes	NP_524760.1
7227	resilin	Late_genes	NP_611157.1
7227	rtv	Late_genes	NP_572693.1
7227	serp	Late_genes	NP_730444.1
7227	stw	Late_genes	NP_610170.2
7227	verm	Late_genes	NP_001163469.1
7227	CCAP-R	Neurostimuli_reception	NP_001368957.1
7227	EHR	Neurostimuli_reception	NP_729905.2
7227	ETHR	Neurostimuli_reception	NP_650960.2
7227	rk	Neurostimuli_reception	NP_476702.1
7227	torso	Neurostimuli_reception	NP_476762.1
7227	Chd64	Sesquiterpenoid_pathway	NP_647860.1
7227	FKBP39	Sesquiterpenoid_pathway	NP_524364.2
7227	JHAMT	Sesquiterpenoid_pathway	NP_609793.2
7227	JHBP	Sesquiterpenoid_pathway	NP_608420.1
7227	JHEBP	Sesquiterpenoid_pathway	NP_611989.1
7227	JHEH	Sesquiterpenoid_pathway	NP_611385.1
7227	met	Sesquiterpenoid_pathway	NP_511126.2
7227	Tai	Sesquiterpenoid_pathway	NP_001245949.1
6689	HR3	Early_genes	XP_027222334.1
6689	dib	Ecdysteroid_pathway	XP_027209672.1
6689	EcR	Ecdysteroid_pathway	XP_027212061.1
6689	E74	Early_genes	XP_027206747.1
6689	E75	Early_genes	XP_027211093.1
6689	Bftz-F1	Early_genes	XP_027236936.1
6689	kkv	Late_genes	XP_027227734.1
6689	knk	Late_genes	XP_027210805.1
6689	sad	Ecdysteroid_pathway	XP_027238669.1
6689	spo	Ecdysteroid_pathway	XP_027213908.1
6689	Usp	Ecdysteroid_pathway	XP_027216942.1
6689	E78	Early_genes	XP_027234687.1
6689	phm	Ecdysteroid_pathway	XP_027215959.1
6689	JHEH	Sesquiterpenoid_pathway	XP_027222530.1
6689	cyp18a1	Ecdysteroid_pathway	XP_027215960.1
6689	JHBP	Sesquiterpenoid_pathway	XP_027223816.1
6689	Chd64	Sesquiterpenoid_pathway	XP_027224334.1
6689	JHEBP	Sesquiterpenoid_pathway	XP_027208769.1
6689	Tai	Sesquiterpenoid_pathway	XP_027215744.1
6689	SREBP	Ecdysteroid_pathway	XP_027238775.1
6689	sro	Ecdysteroid_pathway	XP_027222633.1
6689	HR4	Early_genes	XP_027207778.1
6689	Kr-h1	Fate_genes	XP_027230918.1
6689	nvd	Ecdysteroid_pathway	XP_027231585.1
210409	spo	Ecdysteroid_pathway	XP_045129380.1

210409	sad	Ecdysteroid_pathway	XP_045131452.1
210409	dib	Ecdysteroid_pathway	XP_045105768.1
114398	HR3	Early_genes	XP_042902546.1
114398	EcR	Ecdysteroid_pathway	XP_015926579.1
114398	E74	Early_genes	XP_021001446.2
114398	E75	Early_genes	XP_015930846.1
114398	Bftz-F1	Early_genes	XP_015908511.1
114398	met	Sesquiterpenoid_pathway	XP_042896763.1
114398	shd	Ecdysteroid_pathway	XP_042903873.1
114398	Usp	Ecdysteroid_pathway	XP_015929720.1
114398	E78	Early_genes	XP_015903773.1
114398	JHEH	Sesquiterpenoid_pathway	XP_042906018.1
114398	cyp18a1	Ecdysteroid_pathway	XP_015920197.2
114398	JHAMT	Sesquiterpenoid_pathway	XP_015910904.1
114398	JHEBP	Sesquiterpenoid_pathway	XP_015915401.1
114398	CCAP-R	Neurostimuli_reception	XP_015921275.1
114398	spo	Ecdysteroid_pathway	XP_015926868.1
114398	SREBP	Ecdysteroid_pathway	XP_042907215.1
114398	E93	Fate_genes	XP_042896616.1
114398	HR4	Early_genes	XP_021000290.1
114398	nvd	Ecdysteroid_pathway	XP_042901224.1
7091	cyp15	Sesquiterpenoid_pathway	NP_001140197.1
7070	cyp15	Sesquiterpenoid_pathway	NP_001308597.1

Supplementary Table 4: Curated list of genes involved in the moulting process.

Gene family	Ancestor	Nr of ancestral family duplications	Nr of ancestral family losses
serp;verm	Mandibulata	1	0
serp;verm	Araneae	1	0
kkv;Chs2	Chelicerata	1	0
kkv;Chs2	Pancrustacea	1	0
knk	Arthropoda	2	0
Cda4	Araneae	1	0
ppos	Non tetrapulmonata	0	1
ppos	Acari	0	1
nvd	Coleoptera	0	1
nvd	Mesostigmata	0	1
chinmo	Ostracoda	0	1
chinmo	Malacostraca	0	1
chinmo	Branchiopoda	0	1
E93	Branchiopoda	0	1

Supplementary Table 5: Summary of deep time events from selected gene families.

Total count of duplications inferred by reconciliation and losses inferred by gene prediction assessment are mapped to clade ancestors, as plotted in Figure 5.

Query NCBI identifier	Species	Gene name
NP_001097865.1	Drosophila melanogaster	E93
XP_042896616.1	Parasteatoda tepidariorum	E93
XP_037795760.1	Paeneus monodon	E93
TRY61992.1	Tigriopus californicus	chinmo
XP_015835513.1	Tribolium castaneum	chinmo
NP_609287.3	Drosophila melanogaster	mco1
XP_045026750.1	Daphnia magna	mco1
XP_042224188.1	Hommarus americanus	mco1
XP_042229820.1	Hommarus americanus	ppo1
NP_476812.1	Drosophila melanogaster	ppo1
XP_042903490.1	Parasteatoda tepidariorum	hemocyanin chain A
NP_001097670.1	Drosophila melanogaster	nvd
XP_026296485.1	Apis mellifera	nvd
XP_015795304.1	Tetrachynus urticae	nvd
XP_042901224.1	Parasteatoda tepidariorum	nvd

Supplementary Table 6: Query genes for gene prediction in unannotated assemblies.
